# Home-based high-intensity interval training improves cardiorespiratory fitness: a systematic review and meta-analysis

**DOI:** 10.1186/s13102-023-00777-2

**Published:** 2023-12-05

**Authors:** Katsunori Tsuji, Yosuke Tsuchiya, Hisashi Ueda, Eisuke Ochi

**Affiliations:** 1 Sports Research Center, Hosei University, 4-1 Kizukiomachi Nakahara, Kawasaki, Kanagawa 211-0031 Japan; 2 Center for Liberal Arts, Laboratory of Health and Sports Sciences, Meiji Gakuin University, 1518, Kamikurata-Cho, Totsuka, Yokohama, Kanagawa 244-8539 Japan; 3 Faculty of Health and Medical Science, Teikyo Heisei University, 4-1, Uruidominami, Ichihara, Chiba 290-0193 Japan; 4 Faculty of Bioscience and Applied Chemistry, Hosei University, 3-7-2, Kajino, Koganei, Tokyo 184-8584 Japan; 5 Graduate School of Sports and Health Studies, Hosei University, 4342, Aihara-Cho, Machida, Tokyo 194-0298 Japan

**Keywords:** VO_2_peak, Aerobic capacity, Home-based exercise, High intensity intermittent training, Cardiovascular, Cardiac function

## Abstract

**Background:**

High-intensity interval training (HIIT) is an effective methods to improve maximal oxygen uptake. However, there is no definitive conclusion about the specific effectiveness of home-based HIIT. This review investigated the effects of home-based HIIT on cardiorespiratory fitness in a systematic review and meta-analysis.

**Methods:**

Four electronic databases were searched (PubMed, Cochran database, Web of Science, Igaku Chuo Zasshi) for studies through March 25, 2023. Eligibility criteria include randomized controlled trials of home-based HIIT in adult people regardless disease or handicaped. Comparisons were made between non-exercise controls, laboratory-based HIIT, and moderate-intensity continuous training (MICT). The primary outcome was defined as cardiorespiratory fitness and the secondary outcome was defined as patient-reported outcomes. The standardized mean difference (SMD) with 95% confidence intervals (CIs) was calculated for quantitative indices. The random-effect model was used as the pooling method.

**Results:**

Two hundred seven studies were identified, and 15 satisfied the inclusion criteria. The meta-analysis for cardiorespiratory fitness showed superiority of home-based HIIT to non-exercise controls (SMD 0.61, 95% CI: 0.21, 1.02). However, no significant difference in cardiorespiratory fitness was observed between home-based HIIT and lab-based HIIT (SMD: -0.35, 95%CI: -0.73, 0.03). Also, no significant difference was observed between the home-based HIIT and MICT (SMD 0.34, 95% CI: -0.05, 0.73).

**Conclusion:**

These results indicated that home-based HIIT was an effective intervention for improving cardiorespiratory fitness in healthy adults and patients. Importantly, this review found no significant differences in cardiorespiratory fitness between home-based HIIT and the group of laboratory HIIT and MICT, highlighting its comparable effectiveness and potential as a practical and valuable exercise intervention.

## Background

Cardiorespiratory fitness (CRF) is strongly associated with reduced risk of all-cause mortality, cancer mortality, and cardiovascular morbidity [1–4]. In fact, in a person with low cardiorespiratory fitness, an improvement in peak oxygen uptake (VO_2_peak) of 1 ml/kg/min can reduce the risk of all-cause mortality by 9% [5]. Therefore, training programs are needed for improving cardiorespiratory fitness.

Although moderate-intensity continuous training (MICT) has conventionally been used as an intervention to increase cardiorespiratory fitness, increasing attention is being paid to high-intensity interval training (HIIT) for its ability to increase cardiorespiratory fitness in a time-efficient manner. HIIT is a training protocol consisting of multiple repetitions of short, high-intensity exercise and rest, and has been shown to improve CRF in both non-athletes and athletes [6, 7]. A systematic review has shown that HIIT, despite shorter exercise duration than MICT, improves cardiorespiratory fitness to a greater extent than MICT [6]. However, the need for space, equipment and professional exercise physiologists or instructors has been a challenge in implementing the reported HIIT programmes in the real world [8].

The present systematic review aimed to elucidate the differences between home-based HIIT, non-exercise controls, lab-based HIIT, and MICT. Furthermore, a meta-analysis was conducted to evaluate the efficacy of home-based HIIT compared with non-exercise controls, lab-based HIIT, or MICT on CRF.

## Methods

The Preferred Reporting Items for Systematic Reviews and Meta-Analyses (PRISMA) 2020 checklist was used for this review [9], and the study protocol was registered with PROSPERO (Registration Number: CRD42021260812), an international database of prospectively registered systematic reviews in health and social care.

### Information sources and search strategy

Electronic databases (PubMed, Cochran Library, Web of Science, and Igaku Chuo Zasshi) were searched for studies published using all available records up to March 25, 2023. The following search expression used:

"home*"[Title/Abstract] AND "high-intensity"[Title/Abstract] AND ("interval"[Title/Abstract] OR "intermittent"[Title/Abstract] OR "aerobic interval"[Title/Abstract]) AND (exercise OR training) AND (randomized controlled trial[pt] OR controlled clinical trial[pt] OR randomized[tiab] OR placebo[tiab] OR randomly[tiab] OR trial[tiab] OR intervention[tiab] OR groups[tiab] NOT (animals [mh] NOT humans [mh]))

All studies with keywords related to home-based HIIT interventions were included.

### Inclusion criteria

Inclusion criteria were randomized controlled trials published in English and Japanese with full text available that included home-based HIIT in as an intervention in adult people regardless disease or handicaped. The comparison control groups were lab-based HIIT, home-based or lab-based MICT, or a non-exercise control group. HIIT was defined as exercise consisting of multiple repetitions of short bursts (≤4 min) of high-intensity (≥80% of HRmax, maximal aerobic power, or rating of perceived exertion [RPE] ≥ 15/20 or 6/10) exercise (e.g., weight-bearing exercise, stationary cycling, or use of outdoor/indoor equipment) alternated with low-intensity exercise or passive rest [10, 11]. MICT was defined as exercise consisting of prolonged moderate-intensity (40-70% of HRmax, maximal aerobic power or RPE 11-13) continuous exercise (e.g., weight-bearing exercise, stationary cycling, or treadmill) [12]. The selection criteria for the non-exercise control group were defined as having either no intervention or an intervention that did not improve CRF.

### Study selection and data extraction

Irrelevant articles were excluded from the review by screening the titles and abstracts displayed in the search results (KT and YT). To ensure a consistent understanding of inclusion criteria and data extraction processes among the review team, an experienced systematic reviewer (KT) provided a detailed training session to all co-researchers at the start of the screening process. Additionally, a pilot study was conducted using a sample of the literature to assess and confirm the inter-rater agreement. Following this, intervention methods (exercise duration/frequency, exercise intensity, mode of exercise, HIIT intervals, and intervention setting), study design, and study outcomes (cardiorespiratory fitness, patient reported physical activity and fatigue, adverse events, and compliance) were determined by reviewing the full text of the articles. The full text was independently reviewed by three of the authors (KT, YT, and EO). During the screening process, the evaluations of each author were collated, and the studies deemed eligible were identified through a consensus-based decision. In situations where disagreements arose regarding the inclusion of abstracts and full-text articles, these were resolved by engaging in consensus discussions. When there was a lack of data in certain studies, the corresponding author was contacted for further clarification. To manage the collected data, a researcher meticulously organized all relevant findings from the included studies into an Excel spreadsheet (Microsoft® Excel 2019, Microsoft Corporation). These outcomes were selected to investigate the effects of HIIT on cardiorespiratory fitness as the primary outcome of interest, as well as the effects of HIIT on areas of clinical concern for patients with some illness (patient-reported physical activity and fatigue) and safety of and compliance with HIIT.

### Outcome measures

Cardiorespiratory fitness was evaluated by gas analysis, which enabled the direct measurement of oxygen consumption during exercise. Gas analysis was carried out using methods such as the Douglas bag method or open-circuit spirometry. Treadmill or cycle ergometer were used as exercise modalities for this purpose. To assess patient-reported physical activity, we used a validated questionnaire. The questionnaires were administered at baseline and at the end of the intervention period to assess changes in physical activity levels. To assess patient-reported fatigue, we used a validated questionnaire. The questionnaires were administered at baseline and at the end of the intervention period to assess changes in fatigue levels.

### Risk of bias assessment

The Cochrane risk of bias 1 tool was used to maintain internal validity. All of the authors (KT, YT, HU, EO) assessed selection bias, performance bias, detection bias, attrition bias, reporting bias, and other biases. Other biases were assessed for compliance with the intervention. Any disagreements was resolved through discussion in a video conference.

### Statistical analysis

To determine the pooled effect size of home-based HIIT on VO_2_peak, a random effects meta-analysis was performed using meta-analysis software Review Manager (Version 5.3; The Nordic Cochrane Centre, Copenhagen). Separate analyses were performed to examine the pooled effects of changes in VO_2_peak for home-based HIIT versus non-exercise controls, home-based HIIT versus lab-based HIIT, and home-based HIIT versus MICT. The precision of the pooled effect was expressed as the 95% confidence interval (CI). Effect sizes for continuous variables were calculated as the standardized mean difference (SMD) when different methods were used to assess other outcomes (patient-reported physical activity and fatigue). The treatment effect was calculated as the difference from baseline between the start of the intervention and the end of follow-up. For each outcome, variance was estimated based on the standard deviation of the mean difference. Heterogeneity was assessed using the *I*^*2*^ statistic and was considered significant at p < 0.05. Heterogeneity was considered minimal when *I*^*2*^ was 0% to 30%, moderate when 30% to 50%, substantial when 50% to 90%, and significant when 90% or greater. Publication bias was assessed by examining funnel plot asymmetry with Egger's test, performed using R version 4.2.0 (R Core Team, 2022). A p-value of less than 0.05 was considered indicative of significant publication bias.

## Results

**Fig. 1:**
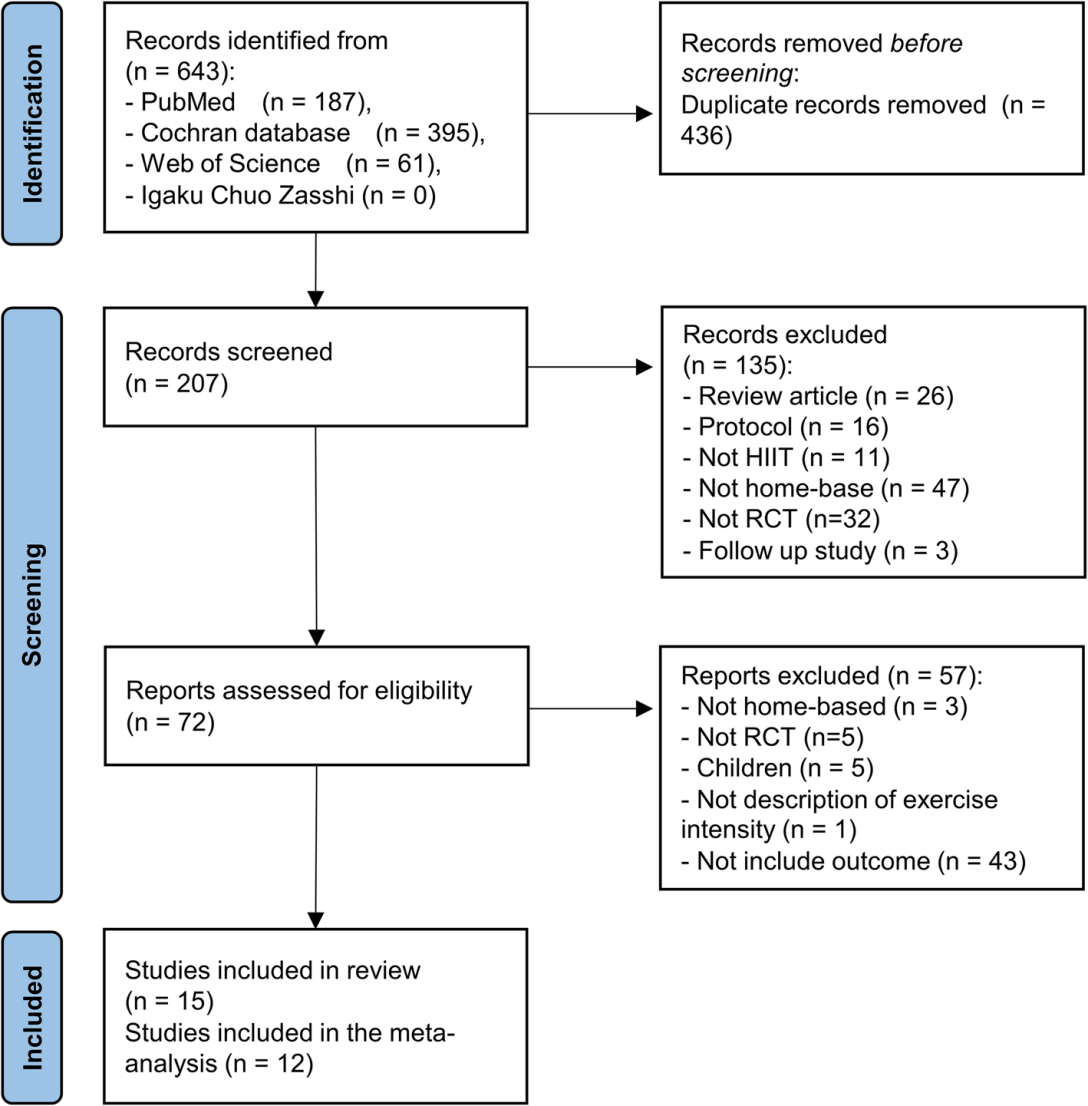
PRISMA 2020 flow diagram

643 articles were obtained from the four databases. 207 duplicates were excluded, and 135 articles were excluded based on screening of their title and abstract. Then, the full text of 72 articles was reviewed and an additional 57 studies were excluded for not meeting the inclusion criteria. Finally, this left a total of 15 studies that satisfied the inclusion criteria. All 15 studies were included in the qualitative systematic review, and 12 studies were included in the quantitative systematic review (Fig. 1).

Tables 1 summarize the studies included in the review. The table lists the authors, sample size, a summary of the HIIT program, outcomes, whether the intervention was supervised or unsupervised, and the intervention setting (home-based HIIT, lab-based HIIT, MICT, or non-exercise control) for each study. Summaries of the HIIT or MICT methods include the duration of training, frequency, mode of exercise, and intensity.

**Table 1:** Summary of included studies characteristics

Study	Participant characteristics	Sample size	Comparison	Duration and frequency (total times)	Mode	Intensity	Interval and recovery durations	Supervised or Unsupervised	Adherence	Monitoring of exercise intensity	Place
Aamot IL, et al. 2013 [13]	coronary artery disease	90 (80 men/10 women)		12 weeks, 2 × /week (24)	up-hill walking, cross country skiing, bicycling, running, or using indoor equipment such as treadmills or cross trainers	85–95% of peak HR (70% of peak HR at active recovery)	4 × 4 min cyclying, 4 min active recovery, total 28 min	combine (two initial sessions with personal instruction of a physiotherapist)	The median is 24 (range: 10–24) sessions	Mean exercise intensity the last 2 min of the intervals was 90 ± 2.5% of peak HR	home environment
			supervised laboratory HIIT	12 weeks, 2 × /week (24)	Treadmill	85–95% of peak HR (70% of peak HR at active recovery)	4 × 4 min cyclying, 4 min active recovery, total 28 min	Supervised by physiotherapist	24 (7–24) sessions	-	hospital
Bankolé LC, et al. 2016 [14]	facioscapulohumeral muscular dystrophy	16		8 weeks, 3 × /week (24)	stationary ergocycle	80% of maximal aerobic power (40% of maximal aerobic power)	5 × 1 min cyclying, 4 min active recovery, total 17 min	combine (The first 5 to 10 training sessions were supervised)	91%	No report	home
			control	-	-	-	-	-	-	-	-
Blackwell J, et al. 2017 [15]	middle-aged healthy adult	18 (5 men/13 women)		4 weeks, 3 × /week (12)	bodyweight-based exercises	the maximum number of repetitions possible with good form during each exertion	5 × 1 min exercise, 90 s active recovery, total 11 min	unsupervised	100%	No report	home
			supervised laboratory HIIT	4 weeks, 3 × /week (12)	stationary ergocycle	95–110% of maximal load (watts (W)) (unloaded cycling at active recovery)	5 × 1 min cyclying, 90 s active recovery, total 11 min	supervised	100%		laboratory
Gauthier C, et al. 2018 [16]	spinal cord injury patients	11		6 weeks, 3 × /week (18)	wheelchair	rating of perceived exertion (RPE) was 6 to 8 (very hard) on a 1 to 10 Borg scale	20 × 30 s exercise, 60 s active recovery, total 30 min	unsupervised	15.5 ± 2.1 sessions	Participants self-reported that they had reached the prescribed exercise intensity	home environment
			MICT	6 weeks, 3 × /week (18)	wheelchair	rating of perceived exertion (RPE) was 4 (somewhat difficult) to 5 (difficult) on a 1 to 10 Borg scale	30 min exercise	unsupervised	17.6 ± 1.7 sessions		home environment
Krawcyk RS, et al. 2019 [17]	mild stroke patient	71		12 weeks, 5 × /week (60)	stationary ergocycle	77–93% of the maximum heart rate, corresponding to 14–16 on the Borg-rated perceived-exertion scale	3 × 3 min exercise, 2 min active recovery, total 13 min	unsupervised	93%	Self-report using a talk test	home
			control	-	-	-	-	-	-	-	-
Taylor JL, et al. 2020 a [18]	coronary artery disease	42 (34 men/8 women)		4 week supervised cardiac rehabilitation program, and followed by 3 × /weekfor 11 months (132)	treadmill or bike	rating of perceived exertion (RPE) was 15 to 18 (hard to very hard) on a 6 to 20 Borg scale	4 × 4-min high-intensity intervals interspersed with 3 min of active recovery	combine (4-week cardiac rehabilitation program were supervised, following 11 months were informal support only)	91% at 3 months 53% over 12 months	The mean RPE was 16.3 ± 1.3, and the mean %HRpeak was 87% ± 6%	home
			MICT	4 week supervised cardiac rehabilitation program, and followed by 3 × /weekfor 11 months (132)	treadmill or bike	34 min of moderate-intensity exercise at an RPE 11 to 13 (fairly light to somewhat hard)	-	combine (4-week cardiac rehabilitation program were supervised, following 11 months were informal support only)	91% at 3 months 41% over 12 months		home
Taylor JL, et al. 2020 b [19]	coronary artery disease	93 (78 men/15 women)		4 week supervised cardiac rehabilitation program, and followed by 3 × /week for 11 months (132)	participants individually in their own environment (e.g. treadmill, cycle ergometer, elliptical machine, rowing ergometer)	rating of perceived exertion (RPE) was 15 to 18 (hard to very hard) on a 6 to 20 Borg scale	4 × 4-min high-intensity intervals interspersed with 3 min of active recovery	combine (4-week cardiac rehabilitation program were supervised, following 11 months were informal support only)	77% at 3 months 56% over 12 months	The mean RPE was 16.5 ± 1.2, and the mean %HRpeak was 88% ± 6%	home
			MICT	4 week supervised cardiac rehabilitation program, and followed by 3 × /weekfor 11 months (132)	treadmill or bike	34 min of moderate-intensity exercise at an RPE 11 to 13 (fairly light to somewhat hard)	-	combine (4-week cardiac rehabilitation program were supervised, following 11 months were informal support only)	86% at 3 months 39% over 12 months		home
Taylor JL, et al. 2021 [20]	coronary artery disease	93 (78 men/15 women)		4 week supervised cardiac rehabilitation program, and followed by 3 × /week for 11 months (132)	outdoor walking exercise or use personal exercise equipment in their home or commercial gym (e.g. bike, treadmill, elliptical)	rating of perceived exertion (RPE) was 15 to 18 (hard to very hard) on a 6 to 20 Borg scale	4 × 4-min high-intensity intervals interspersed with 3 min of active recovery	combine (4-week cardiac rehabilitation program were supervised, following 11 months were informal support only)	77% at 3 months 56% over 12 months	The mean RPE was 16.5 ± 1.2, and the mean %HRpeak was 88% ± 6%	home
			MICT	4 week supervised cardiac rehabilitation program, and followed by 3 × /weekfor 11 months (132)	treadmill or bike	34 min of moderate-intensity exercise at an RPE 11 to 13 (fairly light to somewhat hard)	-	combine (4-week cardiac rehabilitation program were supervised, following 11 months were informal support only)	86% at 3 months 39% over 12 months		home
Mueller S, et al. 2021 [21]	heart failure with preserved ejection fraction	176 (59 men/117 women)		12 months, 3 × /week (144)	stationary ergocycle	80%-90% of maximal heart rate	4 × 4-min intervals interspersed with 3 min of active recovery	combine (3 month supervised followed by 9 month nonsupervised training)	73%	Monitoring with a heart rate sensor (Heart rate values were not reported.)	3 months in clinic followed by 9 months in home
			MICT	12 months, 5 × /week (240)	stationary ergocycle	40 min at 35%-50% of max heart rate reserve	-	combine (3 month supervised followed by 9 month nonsupervised training)	76%		3 months in clinic followed by 9 months in home
Borrega MY, et al. 2021 [22]	healthy men or women aged between 18 and 65 years	67 (22 men/45 women)		6 weeks, 6 × /week (36)	bodyweight-based exercises	85–95% of maximal heart rate	10–12 sets of 30–90 s with 15–60 s of rest between sets	unsupervised	77.78%	No report	home
			MICT	6 weeks, 6 × /week (36)	bodyweight-based exercises	6–8 min at 70–85% max heart rate × 3 sets	1–2 min	unsupervised	80.64%		home
McDonough DJ, et al. 2021 [23]	helthy young adults aged between 18 and 35 years	64 (16 men/48 women)		12 weeks, 1 × /week (12)	bodyweight-based exercises with YouTube-delivered physical activity	unspecified (exercise duration was 6.3 ± 3.9 min)	unspecified	unsupervised	96.90%	No report	home
			control	-	-	-	-	-	-	-	-
Papadopoulos E, et al. 2021 [24]	post cancer on active surveillance men	18		8 weeks, 2 × /week (16)	recubent bike	> 85% of maximal heart rate	10 × 1-min high-intensity intervals interspersed with 1 min of active recovery	supervised	95%	The mean %HRpeak was 86% ± 4%	home
			resistance training	8 weeks, 2 × /week (16)	using strength training apparatus and free weights	3 sets of 8 repetitions at 65% 1 RM	-	supervised	96%		home
Ochi E, et al. 2021 [25]	stage I–IIa breast cancer	44 women		12 weeks, 3 × /week (36)	bodyweight-based exercises	rating of perceived exertion (RPE) was 18 ± 2 on a 6 to 20 Borg scale	8 sets of 20 s exercise with 10 s rest	combine (For the first 6 weeks, there was 1 subervised per week)	86% (range 19%–100%)	The mean RPE was 18 ± 2	home
			control	-	-	-	-	-	-	-	-
Sian TS, et al. 2021 [26]	healthy young adults aged between 18 and 30 years	30 (15 men/15 women)		4 weeks, 3 × /week (12)	bodyweight-based exercises	> 85% of maximal heart rate	5 sets of 60 s exercise with 90 s rest	unsupervised	100%	100% (% of training sessions where the target heart rate threshold of 85% HRmax was achieved)	home
			supervised laboratory HIIT control	4 weeks, 3 × /week (12) -	cycle ergometer -	> 85% of maximal heart rate -	5 sets of 60 s exercise with 90 s rest -	supervised -	100%		home
Sian TS, et al. 2022 [27]	sedentary older adults	30 (14 men/16 women)		4 weeks, 3 × /week (12)	bodyweight-based exercises	> 85% of maximal heart rate	5 sets of 60 s exercise with 90 s rest	unsupervised	100%	100% (% of training sessions where the target heart rate threshold of 85% HRmax was achieved)	home
			supervised laboratory HIIT control	4 weeks, 3 × /week (12) -	cycle ergometer -	> 85% of maximal heart rate -	5 sets of 60 s exercise with 90 s rest -	supervised -	100%		home

### Risk of bias assessment

**Fig. 2:**
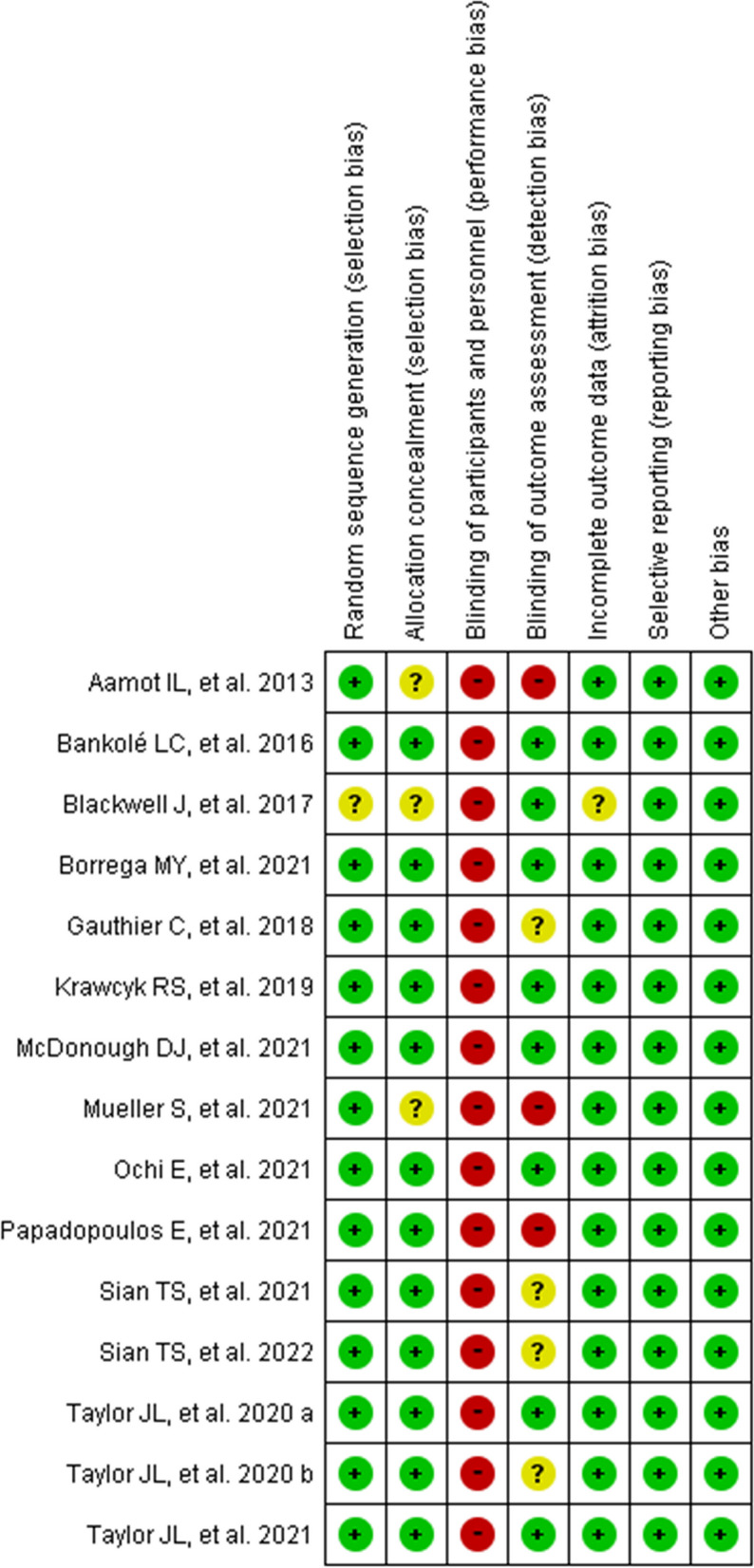
Summary of the Cochrane risk of bias tool

The results of the methodological quality assessment of the studies included in this review are summarized in Fig. 2. The proper procedure for randomly generated sequences was fully described in 15 studies, 1 of which hid the assignments. Performance bias was found in all included trials. Blinding of participants was not possible due to the nature of the exercise interventions, though this does not pose a threat in terms of internal validity. Eight studies blinded outcome assessors and 3 studies did not.

### Setting (home- or lab-based HIIT, MICT, and control)

Five studies involved interventions for healthy adults [15, 22, 23, 26, 27] and 10 involved interventions for patients [13, 14, 16–21, 24, 25]. The comparison group versus home-based HIIT was lab-based HIIT in 4 studies [13, 15, 26, 27], MICT in 7 [14, 16, 18–22], resistance training in 1 [24], and non-exercise controls in 3 studies [17, 23, 25]. The intervention was supervised in 2 studies [17, 24], unsupervised in 6 [15, 16, 22, 23, 26, 27], and a combination of supervised and unsupervised in 7 [13, 14, 18–21, 25]. The exercise modality for home-based HIIT included 6 weight-bearing exercise [15, 22, 23, 25–27], 6 using stationary cycling or a treadmill [14, 17–19, 21, 24], and 3 using outdoor/indoor equipment [13, 16, 20].

### Cardiorespiratory fitness: home-based HIIT versus non-exercise controls

**Fig. 3:**
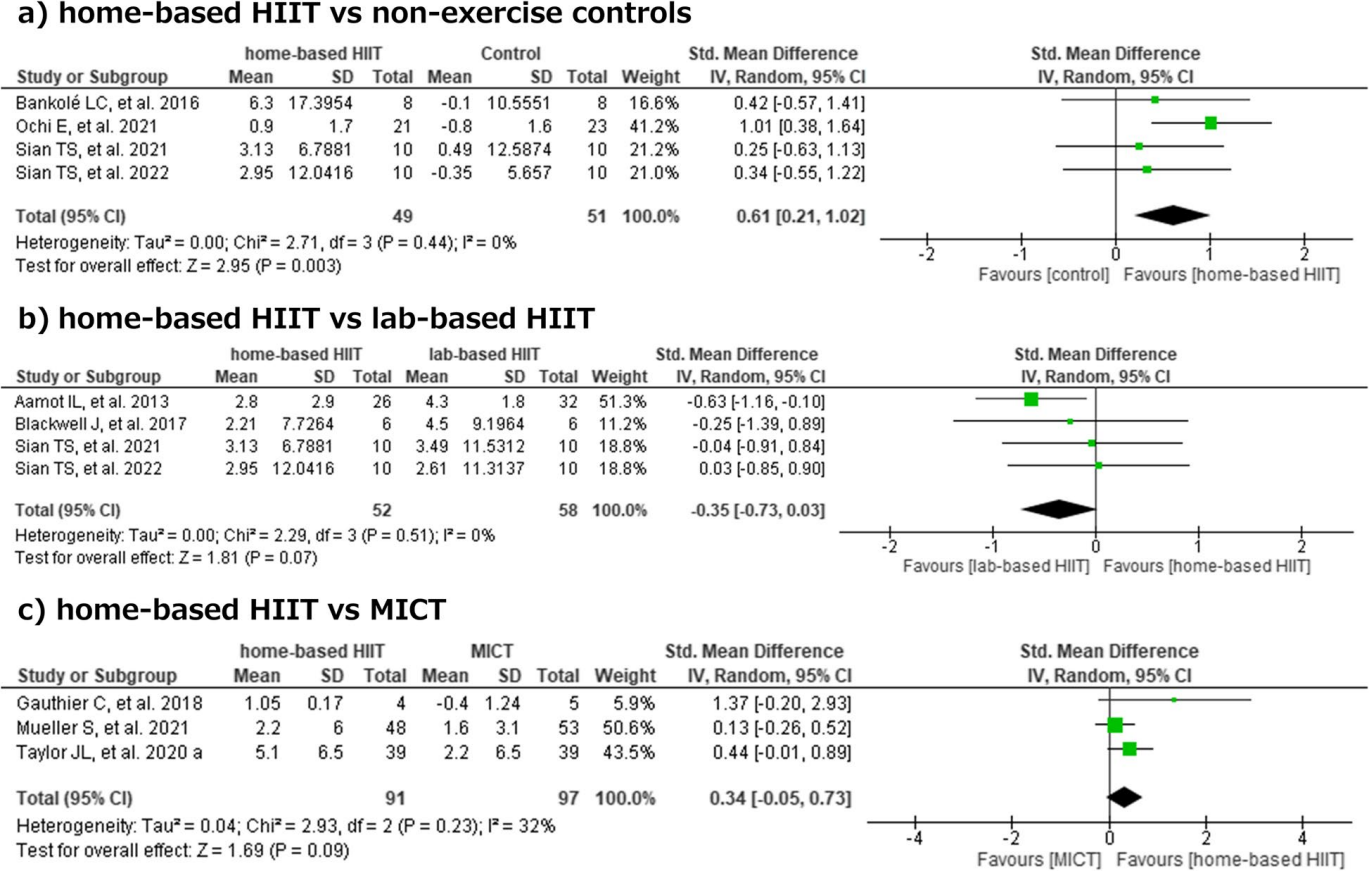
Effects of home-based HIIT vs no-exercise controls or lab-based HIIT or MICT on cardiorespiratory fitness. CL: confidence limits, HIIT: high-intensity interval training, MICT: moderate intensity continuous training

Four studies with a total of 100 participants compared home-based HIIT versus non-exercise controls (Fig. 3a) [14, 25–27]. Compared with the non-exercise control group, the meta-analytic effects of home-based HIIT appeared to provide a greater benefit in terms of VO_2_peak (SMD 0.61; 95% CI [0.21, 1.02]; p = 0.003). Egger's test suggested potential publication bias, though not statistically significant (intercept = -4.239, 95% CI -6.75—-1.73, t = -3.316, p = 0.080). However, a visual examination of the funnel plot showed symmetry, indicating no evident bias.

### Cardiorespiratory fitness: home-based HIIT versus lab-based HIIT

Four studies with a total of 110 participants compared home-based HIIT and lab-based HIIT (Fig. 3b) [13, 15, 26, 27]. No significant difference in VO_2_peak was observed between home-based HIIT and lab-based HIIT (SMD -0.35; 95% CI [-0.73, 0.03]; I^2^ = 0%; p = 0.07). Egger's test indicated no significant evidence of publication bias in the meta-analysis (intercept = 2.175, 95% CI 0.07—4.28, t = 2.026, p = 0.180). The funnel plot appeared symmetrical upon visual inspection, aligning with the Egger's test results.

### Cardiorespiratory fitness: home-based HIIT versus MICT

Three studies with a total of 188 participants compared home-based HIIT versus MICT (Fig. 3c) [16, 18, 21]. No significant difference in VO_2_peak was observed between home-based HIIT and MICT (SMD 0.34; 95% CI [-0.05, 0.73]; I^2^ = 32%; *p* = 0.09). Egger's test did not reveal any statistical evidence of publication bias (intercept = 2.236, 95% CI -0.05—4.53, t = 1.914, p = 0.307). Visual inspection of the funnel plot suggested symmetry, supporting the absence of publication bias.

### Patients-reported outcomes

**Fig. 4:**
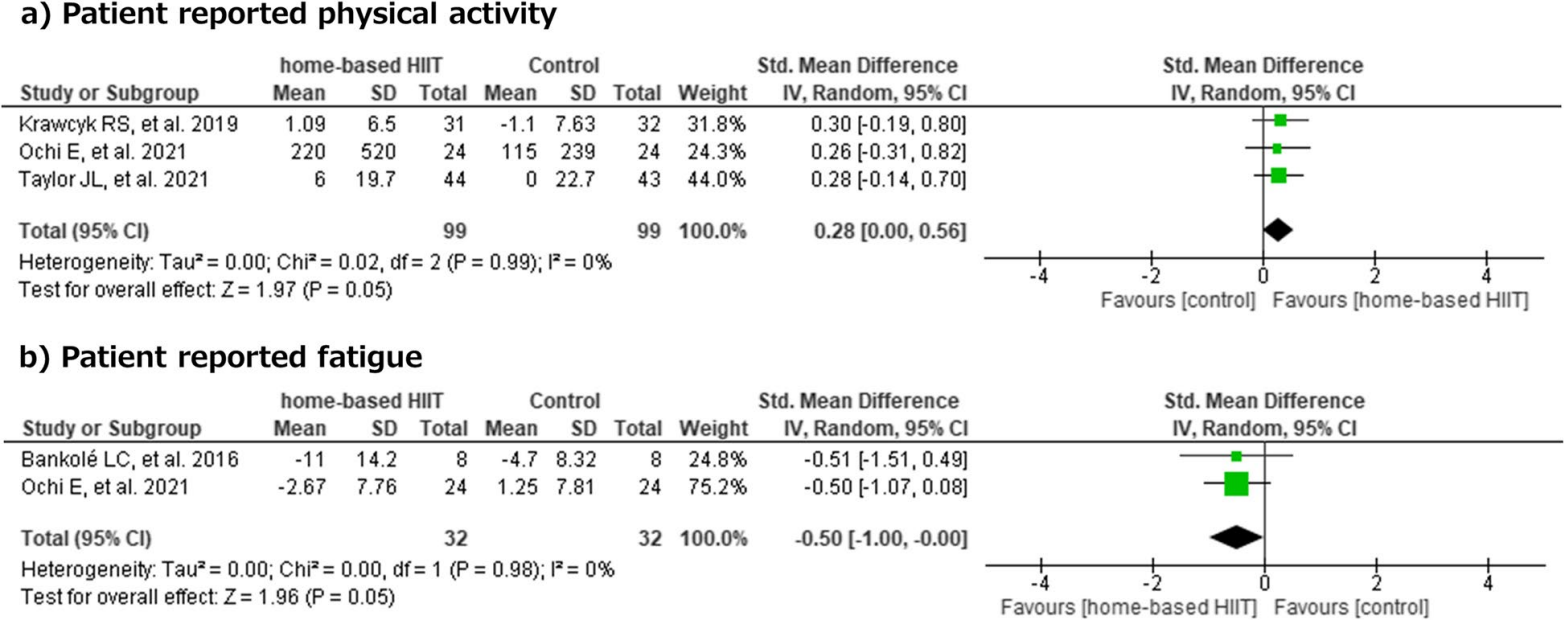
Effects of home-based HIIT vs no-exercise controls on physical activity and patient reported fatigue. CL: confidence limits, HIIT: high-intensity interval training

Patient-reported physical activity was analyzed in 3 studies with a total of 198 participants. No significant difference in physical activity was observed between the home-based HIIT group and the non-exercise control group (SMD 0.28; 95% CI [0.00, 0.56]; I^2^ = 0%; p = 0.05) (Fig. 4a) [17, 25]. Patient-reported fatigue was analyzed in 2 studies with a total of 64 participants. No significant difference in fatigue was observed between the home-based HIIT group and the non-exercise control group (SMD -0.50; 95% CI [-1.00, 0.00]; I^2^ = 0%; p = 0.05) (Fig. 4b) [14, 25].

### Compliance rate and adverse events

All studies (15/15; 100%) reported exercise program compliance rates. The mean rate of exercise program compliance across all studies was 80% (range: 35–100%). Five of the studies also reported heart rate during exercise, with an average value of 87% HRmax.

Of the 15 studies included in this systematic review, all of the Taylor et al. studies (20–22) had the same study participants, so we reviewed what was reported in 13 studies. All trials (13/13; 100%) commented on adverse events in the exercise interventions, but 10 trials did not state that they used a specific protocol for collecting adverse events. No cardiac-related events resulting in death or hospitalization during training occurred in any of the intervention groups among the studies that reported events. The four studies that reported adverse events included exercise-induced shoulder pain [16], hypotension during exercise [18], post-exercise cardiovascular events (obstruction due to bypass graft failure) [21], and serious events occurred in patients with coronary artery disease [20]. No adverse events occurred in the other 10 studies.

## Discussion

The major findings of this review were that 1) the CRF of home-based HIIT is more effective than that of no-exercise control, 2) home-based HIIT is as effective as lab-based HIIT in increasing cardiorespiratory fitness, and 3) home-based HIIT is as effective as MICT in increasing cardiorespiratory fitness. These points are discussed below.

### *Home-based HIIT versus non-exercise control in VO*_*2*_*peak*

Meta-analysis of the Four studies that compared home-based HIIT and non-exercise control groups showed that the standard mean difference (SMD) and mean difference (MD) in VO_2_peak between the groups were 0.61 (95% CI: 0.21, 1.02) and 1.75 ml/kg/min (95% CI: 0.79, 2.72), respectively. Since we were able to standardize the rating scale for all of the studies included in this study, we discuss the results based on mean differences. VO_2_peak is strongly associated with all-cause mortality risk; a 1 ml/kg/min improvement in VO_2_peak is associated with a 9% reduction in all-cause mortality risk [5]. In addition, a 10% increase in VO_2_peak is customarily used in determining the effectiveness of exercise programs [28].The pre-intervention VO_2_peak of the participants included in this systematic review ranged from 25.0 to 36.1 ml/kg/min [17, 23, 25], suggesting that home-based HIIT is an effective method for improving VO_2_peak.

### *Home-based HIIT versus lab-based HIIT in VO*_*2*_*peak*

The present systematic review directly compared the effects of home-based HIIT and lab-based HIIT. Meta-analysis of the four studies that compared home-based HIIT and lab-based HIIT showed that the SMD and MD in VO_2_peak between groups were -0.35 (95% CI: -0.73, 0.03) and -1.46 ml/kg/min (95% CI: -2.70, -0.22), respectively. The results based on the standardized mean difference clearly indicate that home-based HIIT protocols are as effective as lab-based HIIT, which is a pivotal point we wish to emphasize in this paper. This significant finding highlights the potential of home-based HIIT as a feasible alternative to lab-based protocols. In a study by Menz et al., which was not included in the present systematic review, HIIT (unclear whether home-based or not) performed with weight-bearing exercises (VO_2_peak: pre: 49.5 ± 6.6 ml/kg/min, post: 54.4 ± 5.3 ml/kg/min, p < 0.001) was as effective as HIIT performed on a treadmill (pre: 47.8 ± 5.6 ml/kg/min, post: 54.1 ± 5.6 ml/kg/min, p < 0.001) in increasing VO_2_peak [29]. Therefore, depending on the methodology, home-based HIIT can be as effective as lab-based HIIT in increasing VO_2_peak. Home-based HIIT is notably accessible, requiring little or no specialized equipment and adaptable to various domestic settings. Its suitability for short, effective workouts adds to its practicality. Moreover, integrating it into an online framework improves monitoring feasibility. To further enhance the effects of home-based HIIT, it is necessary to establish methods such as online interventions, monitoring, and feedback, aimed at improving adherence to HIIT, increasing the intensity of exercise, and maintaining a high exercise intensity for a longer duration.

### *Home-based HIIT versus MICT in VO*_*2*_*peak*

Meta-analysis of the three studies that compared home-based HIIT and MICT showed that the SMD and MD in VO_2_peak between groups were 0.34 (95% CI: -0.05, 0.73) and 1.40 ml/kg/min (95% CI: 0.50, 2.30), respectively. This indicates that home-based HIIT may be as effective as MICT in improving cardiorespiratory fitness. Notably, our review included studies involving diverse populations, such as athletes and individuals with medical conditions, suggesting the potential safety and effectiveness of home-based HIIT across various groups [16, 18, 21]. For patients with health conditions, this underscores the potential value of HIIT as an at-home exercise therapy. Importantly, all instances of MICT in the studies reviewed were specifically home-based, aligning directly with our focus on home-based HIIT. This further underscores our finding that home-based HIIT and home-based MICT demonstrate comparable effectiveness in improving cardiorespiratory fitness. Milanovic et al. conducted a meta-analysis of 19 studies comparing the effect of lab-based HIIT on VO_2_peak with that of MICT and found that lab-based HIIT was more effective than MICT in improving VO_2_peak, with an MD of 1.2 ± 0.9 ml/kg/min. Hannan et al. conducted a systematic review and meta-analysis of the impact of lab-based HIIT and MICT on VO_2_peak in patients with coronary artery disease [30] and found that lab-based HIIT provided a greater benefit than MICT, with a SMD in VO_2_peak of 0.43 (95% CI: 0.23, 0.62). They also found that HIIT was as safe as MICT for patients undergoing cardiac rehabilitation. A definitive conclusion cannot be reached as to whether home-based HIIT can be an alternative to MICT for improving cardiorespiratory fitness because of the small number of studies to date and the lack of established methodology for home-based HIIT. Nevertheless, the results of this study, showing that short-duration home-based HIIT may be as effective as long-duration MICT in improving cardiorespiratory fitness, which leads saving time, appears to provide very important information for exercise prescription.

### Other outcomes

#### Patients reported outcomes (Fig. 4)

Only a few studies have evaluated the effects of home-based HIIT on physical activity and fatigue in patients. The results of these studies showed that home-based HIIT provided less beneficial effects on physical activity and fatigue compared to non-exercise control groups [14, 25]. However, there was a trend toward beneficial effects of home-based HIIT in both outcomes, warranting further studies in patients with physical inactivity and fatigue.

#### Compliance rate and adverse events

All studies (13/13) reported the rate of compliance with home-based HIIT, with a mean of 80% (range: 35–100%). Although the average compliance rate was high, looking at individual studies, one study had a compliance rate of as low as 35%, suggesting that interventions are needed to further increase the compliance rate. In particular, when HIIT is performed at home and non-supervised, interventions such as exercise motivation, monitoring of exercise intensity, and monitoring of movement posture need to be provided to the exerciser even in the absence of a supervisor.

All studies (15/15) commented on adverse events, with 10 studies reporting the absence of adverse events. In a study of wheelchair-based HIIT, some participants dropped out due to exercise-induced shoulder pain [16]. A study of patients with coronary artery disease reported one post-exercise event (hypotension) in the HIIT group [18]. In a study of patients with heart failure with preserved ejection fraction, multiple cardiovascular events occurred, but none of them were described as definitely related to the exercise [21]. These results suggest that prescribing home-based HIIT, at least for healthy individuals, is very safe. However, when prescribing home-based HIIT for a patient, it may be necessary to consider the patient's medical condition and seek the judgment of the attending physician, if necessary.

### Limitations

Limitations of the present systematic review include the small number of previous studies included in the meta-analysis, variability in the frequency and duration of the exercise interventions, and the variable or unclear exercise intensity in some studies. Additionally, there was a scarcity of studies demonstrating favorable effects of home-based HIIT. The overall effect size was derived from a limited number of studies (k), with particular concern arising from a single study contributing to over 50% of the weight in the analysis. This poses significant limitations to the interpretation of our results. Furthermore, the limited number of available studies constrained the precision of our heterogeneity [31] and publication bias assessments, impacting the overall reliability of these evaluations. Currently, there is no consensus on the intensity, frequency, and duration of HIIT among researchers, warranting the development of more effective methods for implementing home-based HIIT and further studies. The aforementioned factors should be carefully considered when interpreting the findings of this meta-analysis.

### Implications for home-based HIIT as an intervention

This systematic review and meta-analysis demonstrated the effectiveness of home-based HIIT in improving cardiorespiratory function. Specifically, it was found that the improvement in cardiorespiratory fitness was significantly greater when compared to a no-exercise control group and were similar in magnitude when compared to home-based MICT. However, further studies are needed to explore effective methods for setting and managing exercise intensity in home-based HIIT. It would be desirable, for example, to develop and utilize mobile applications equipped with functions such as monitoring exercise intensity, motivating people to improve compliance with the exercise, and maintaining posture during the exercise. The finding that home-based HIIT was as effective as home-based MICT in increasing cardiorespiratory fitness is extremely important and should be disseminated and implemented promptly.

## Conclusion

The present systematic review and meta-analysis demonstrated that home-based HIIT improves cardiorespiratory fitness. In addition, its effectiveness could potentially be comparable to lab-based HIIT and MICT in improving cardiorespiratory fitness. It is important to recognize that this conclusion is tentative, given the limited number of studies and the need for more comprehensive methodological research. We believe that this understanding is crucial for clinicians, patients, and exercise instructors as it underscores the potential value and applicability of home-based HIIT.

## Data Availability

Not applicable.
